# The effect of AUT00206, a Kv3 potassium channel modulator, on dopamine synthesis capacity and the reliability of [^18^F]-FDOPA imaging in schizophrenia

**DOI:** 10.1177/02698811221122031

**Published:** 2022-09-26

**Authors:** Ilinca Angelescu, Stephen J Kaar, Tiago Reis Marques, Faith Borgan, Mattia Veronesse, Alice Sharman, Anil Sajjala, Bill Deakin, John Hutchison, Charles Large, Oliver D Howes

**Affiliations:** 1 Institute of Psychiatry, Psychology and Neuroscience, King’s College London, London, UK; 2 Max Planck UCL Centre for Computational Psychiatry and Ageing Research, Institute of Neurology, London, UK; 3 Faculty of Medicine, Institute of Clinical Sciences, Imperial College London, London, UK; 4 Department of Information Engineering, University of Padua, Padua, Italy; 5 Autifony Therapeutics Limited, Stevenage Bioscience Catalyst, Stevenage, UK; 6 Division of Neuroscience and Experimental Psychology, University of Manchester, Manchester, UK

**Keywords:** F-DOPA, reliability, test–retest, schizophrenia, Kv3, AUT00206

## Abstract

**Background:**

Current treatments for schizophrenia act directly on dopamine (DA) receptors but are ineffective for many patients, highlighting the need to develop new treatment approaches. Striatal DA dysfunction, indexed using [^18^F]-FDOPA imaging, is linked to the pathoetiology of schizophrenia. We evaluated the effect of a novel drug, AUT00206, a Kv3.1/3.2 potassium channel modulator, on dopaminergic function in schizophrenia and its relationship with symptom change. Additionally, we investigated the test–retest reliability of [^18^F]-FDOPA PET in schizophrenia to determine its potential as a biomarker for drug discovery.

**Methods:**

Twenty patients with schizophrenia received symptom measures and [^18^F]-FDOPA PET scans, before and after being randomised to AUT00206 or placebo groups for up to 28 days treatment.

**Results:**

AUT00206 had no significant effect on DA synthesis capacity. However, there was a correlation between reduction in striatal dopamine synthesis capacity (indexed as Ki^cer^) and reduction in symptoms, in the AUT00206 group (*r* = 0.58, *p* = 0.03). This was not observed in the placebo group (*r* = −0.15, *p* = 0.75), although the placebo group may have been underpowered to detect an effect. The intraclass correlation coefficients of [^18^F]-FDOPA indices in the placebo group ranged from 0.83 to 0.93 across striatal regions.

**Conclusions:**

The relationship between reduction in DA synthesis capacity and improvement in symptoms in the AUT00206 group provides evidence for a pharmacodynamic effect of the Kv3 channel modulator. The lack of a significant overall reduction in DA synthesis capacity in the AUT00206 group could be due to variability and the low number of subjects in this study. These findings support further investigation of Kv3 channel modulators for schizophrenia treatment. [^18^F]-FDOPA PET imaging showed very good test–retest reliability in patients with schizophrenia.

## Introduction

Schizophrenia is a common and severe mental illness with a lifetime prevalence of approximately 0.7% (McCutcheon et al., 2019). It is characterised by positive psychotic symptoms, such as delusions and hallucinations, negative symptoms, such as social withdrawal and amotivation, and impairment in cognitive domains, including attention, working memory, verbal learning and executive function (McCutcheon et al., 2020b). It is a leading cause of adult disease burden (Whiteford et al., 2013) with substantial healthcare and societal costs (Howes and Murray, 2014), which approach 100 billion Euros per year across Europe (Gustavsson et al., 2011). It has been ranked 12th in the top global causes of disability for the last decade (Vos et al., 2016).

Dysregulated striatal dopamine (DA) function is thought to contribute to the development of schizophrenia (Abi-Dargham, 2017; Howes et al., 2015; McCutcheon et al., 2019). Several, [^18^F]-FDOPA PET imaging studies have reported increased striatal dopamine synthesis capacity in patients with schizophrenia, with large effect sizes (Howes et al., 2011a, 2011b; McCutcheon et al., 2019; Reith et al., 1994). Meta-analysis suggests that increases are localised to the associative striatum; a striatal region connected to associative cortical regions such as the dorso-lateral prefrontal cortex (McCutcheon et al., 2018). Studies report that greater striatal DA synthesis capacity in the associative striatum directly correlates with more severe symptoms, predicts greater improvement with a D2 receptor blocking drugs, and is increased in patients with prodromal symptoms (Howes et al., 2011a, 2011b; Jauhar et al., 2017, 2019b). These findings suggest that DA synthesis capacity in the associative striatum measured using [^18^F]-FDOPA PET may be a useful biomarker of the pathophysiology of schizophrenia with predictive potential for treatment selection (Bose et al., 2008). However, while [^18^F]-FDOPA imaging shows good test–retest reliability in healthy volunteers (Egerton et al., 2010), its reliability in patients with schizophrenia is not known. In view of this, one of the study aims was to determine the test–retest reliability of [^18^F]-FDOPA PET imaging in patients with schizophrenia.

Current pharmacological treatments for schizophrenia all act on D2 receptors, with the main effect being the reduction of positive symptoms (Miyamoto et al., 2012). They have little effect on negative and cognitive symptoms, and approximately 30% of patients show only partial benefit (Howes et al., 2017). Moreover, they act downstream of the pathophysiological increase in DA synthesis and release capacity (Howes et al., 2012), and, perhaps as a result, are not able to normalise this underlying abnormality (Howes et al., 2013; Jauhar et al., 2019a). This highlights the need for new therapeutic strategies.

It has been hypothesised that deficits in the function of GABA interneurons, particularly fast-spiking, parvalbumin (PV)-positive interneurons, may lead to the observed dopaminergic dysfunction, which in turn contributes to the symptoms of schizophrenia (Grace, 2016; Kaar et al., 2019; Lewis et al., 2012; McCutcheon et al., 2020a). Supporting this, psychotomimetic doses of drugs, such as ketamine and PCP, that block NMDA receptors expressed on GABA interneurons and which, when given chronically, reduced levels of PV in animals (Neill et al., 2010; Pratt et al., 2008), and, when given acutely, induce psychotic and negative symptoms in healthy people and worsen symptoms in patients with schizophrenia (Abdallah et al., 2018; Beck et al., 2019, 2020; Haaf et al., 2018; Thiebes et al., 2017). Moreover, ketamine elevates striatal DA synthesis capacity in mice, and this effect can be blocked by selectively activating PV-positive interneurons (Kokkinou et al., 2020). Post-mortem studies of brains from patients with schizophrenia show GABA-related alterations, especially for the specific subpopulation of PV-positive interneurons (Berretta et al., 2015; Chung et al., 2016; Dong et al., 2015; Enwright et al., 2016; Schubert et al., 2015), with a reduction in measures of PV seen on meta-analysis (Kaar et al., 2019), and lower levels of GABA markers seen in vivo (Frankle et al., 2015; Marques et al., 2020). GABA interneurons, particularly PV-positive cells, are regulated by Kv3.1 and Kv3.2 potassium channels, which are highly expressed in corticolimbic brain networks (Yanagi et al., 2014). Kv3.1 protein and mRNA expression were reduced in a post-mortem study in a cohort of patients with schizophrenia (Lewis et al., 2012; Yanagi et al., 2014). The reduction in Kv3.1 was only seen in patients that had not been on antipsychotic medication at the time of death. Kv3.1 channels are also expressed by neurons in the basal ganglia, which can regulate DA neuron firing (Parekh et al., 2018). Activation of Kv3.1 channels is proposed to enhance the firing of GABA neurons in these systems to increase inhibition of DA neurons in the basal ganglia and restore the balance of excitation–inhibition in higher cortical circuits (Parekh et al., 2018). Modulation of Kv3.1 channels may therefore provide a novel approach to restore the balance of activity within the DA system and thus reduce symptoms in patients with schizophrenia.

A series of novel, selective modulators of Kv3.1 and Kv3.2 channels has been identified, which can enhance the activity of PV-positive interneurons in vitro (Andrade-Talavera et al., 2020; Boddum et al., 2017; Rosato-Siri et al., 2015). In a study in mice, one of these compounds, AUT1, reduced amphetamine-induced hyperactivity, similar to the antipsychotic clozapine (Parekh et al., 2018). Moreover, AUT1 significantly reduced the firing rate of DA neurons (Parekh et al., 2018). AUT00206, similar to AUT1 (Rosato-Siri et al., 2015), modulates the voltage-dependence of activation of Kv3.1 and Kv3.2 ion channels. The compound lowers the voltage at which these channels are activated and thus facilitates channel opening at more hyperpolarised potentials (Autifony, unpublished data). Consistent with these effects on Kv3.1 and Kv3.2 channels, studies in animal models have shown that AUT00206 can enhance the activity of PV-positive interneurons to rescue gamma frequency cortical and hippocampal network synchrony in vitro (Large et al., 2017), and impaired cognitive function in vivo (Leger et al., 2015). Cross-screening studies indicate that AUT00206 is highly selective for Kv3 channels, with little or no effect on other ion channels, receptors or enzymes examined (Autifony, unpublished data).

AUT00206 has been shown to influence the firing of PV-positive interneurons in vitro and rescue deficits in cognitive and PV expression in rats previously treated with PCP (Lodge, 2017). In a recent clinical study, AUT00206 post-treatment reduced the effect of ketamine on the fMRI BOLD signal in healthy human volunteers (Deakin et al., 2019). In view of this evidence, we aimed to test whether AUT00206 could influence DA synthesis capacity in patients with schizophrenia, measured using [^18^F]-FDOPA PET, and whether this might relate to clinical change (Hutchison et al., 2019). We tested the hypothesis that AUT00206 would reduce DA synthesis capacity in the associative striatum and that greater reduction would be correlated with reduction in total symptom severity measured with the PANSS.

## Materials and method

### Participants

Twenty-four volunteers were recruited from psychosis early intervention services in London, England. The inclusion criteria were as follows: (1) male gender (due to the absence of safety data for AUT02006 in females), (2) diagnosis of schizophrenia as determined by the Diagnostic and Statistical Manual of Mental Disorders-5 (DSM-5) using the Stuctured Clinical Interview for DSM-5 (SCID-5) (First, 2016), (3) able to give informed consent, (4) within 5 years of illness diagnosis, (5) currently stable with no evidence of relapse within the last 2 months prior to study enrolment, (6) currently taking a stable dose of antipsychotic medication for at least 1 month (or had received at least three injections at the same dose if on depot), (7) symptom severity on the Positive and Negative Syndrome Scale (PANSS) (Kay et al., 1987) of at least three on two items of both the positive and negative symptom scales or severity of at least four on one item in the positive, and (8) agreeing to use contraceptive measures for the duration of the trial.

The main exclusion criteria were as follows: (1) current or prior use of clozapine, (2) being severely underweight (body mass index (BMI) under 17.5) or morbidly obese (BMI over 35), (3) positive for HIV or hepatitis B or C, (4) positive for illicit drug use on urine drug test, (5) sensitivity to the study medication or placebo constituents, (6) history of significant adverse reactions to other drugs, (7) history of epilepsy or seizures, (8) type 1 or 2 diabetes, (9) suicidal or homicidal ideation or behaviour, (10) participation in a clinical trial within the last 30 days, (11) history of alcohol or drug dependence in the past year, (12) moderate depression or anxiety indicated by a score of ⩾11 out of 21 on the Hospital Anxiety and Depression Scale (HADS) and (13) contraindications to imaging. Treatment resistance was not an exclusion criterion.

### Design

This present report describes the design, implementation and results for the [^18^F]-FDOPA PET biomarker measurements, conducted as part of a study to explore the safety, tolerability and pharmacokinetics of AUT00206 in patients with schizophrenia stabilised on up to two antipsychotic medications (EudraCT: 2016-002704-63). A placebo group was included for drug safety monitoring, not for a formal comparison of imaging biomarkers with placebo. Side effects and potential adverse events will be published in a separate report and are beyond the scope of the present work.

The aims for the [^18^F]-FDOPA biomarker exploration were (1) to determine whether AUT00206 could alter DA synthesis capacity in these patients, (2) to determine whether any change in DA synthesis capacity might be related to changes in symptom measured with the PANSS and (3) to evaluate the test–retest reliability of [^18^F]-FDOPA imaging in patients with schizophrenia. Our prior studies have shown that DA synthesis capacity in the associative striatum is elevated in patients with schizophrenia with an effect size of 1.1 to 1.3 (Howes et al., 2013; Jauhar et al., 2019a). A power calculation using G*power 3.1 (Faul et al., 2009) indicated that a sample size of 12 would have greater than 90% power to detect a significant reduction due to AUT00206 of this magnitude using a paired *t*-test (*p* < 0.05 two tailed) and a sample size of seven would have greater than 80% power to detect an intraclass correlation coefficient (ICC) of >0.8 in the placebo group. Subjects were scanned at baseline and were then randomised on a 2:1 basis to active treatment or placebo groups. They received AUT00206 or placebo treatment for up to 28 consecutive days. Treatment was added to their current antipsychotic medication, which remained unchanged for the duration of the study. Subjects in the AUT00206 treatment group received a single loading dose of 2000 mg of AUT00206 on day 1, followed by twice-daily doses of 800 mg (1600 mg/day) and a single 800 mg dose on day 28. The placebo group received a matching loading dose of placebo followed by twice daily doses of placebo and a single placebo dose on the final day. The initial loading dose was used to ensure plasma levels of AUT00206 in the target therapeutic range, based on preclinical data, were reached within the first 24 h (data on file; Autifony Therapeutics Ltd, Stevenage, UK). Pharmacokinetic data in healthy volunteers shows that AUT00206 at 1600 mg/day reaches steady-state blood levels by 7 days (data on file; Autifony Therapeutics Ltd). Subjects underwent two [^18^F]-FDOPAPET scans, one at baseline within 28 days prior to the commencement of AUT00206 and the second scan while on-treatment, after a minimum of 14 days of treatment when they had been at steady-state levels for at least 7 days. The study was approved by the NHS research ethics committee and was conducted in accordance with the declaration of Helsinki 1964; all subjects gave written informed consent to participate.

### Clinical measures

Psychiatric assessments included the PANSS, the HADS, the Clinical Global Impressions Scale and the Columbia-Suicide Severity Rating Scale. All patients received these measures at baseline and at day 28 on-treatment (or at dropout if earlier).

### DA synthesis capacity: [^18^F]-FDOPA PET imaging

All participants were asked to fast (except water) 12 h before the scan. Imaging data were collected on a Siemens Biograph 6 HiRez PET scanner (Siemens, Erlangen, Germany) in 3D mode in line with our standard procedure (Egerton et al., 2010; Howes et al., 2009). Subjects received 400 mg entacapone (a peripheral catechol-omethyl-transferase inhibitor) and 150 mg carbidopa (a peripheral aromatic acid decarboxylase inhibitor) orally 1 h before the scan to prevent the formation of radiolabelled metabolites that might cross the blood–brain barrier (Cumming et al., 1993). Participants were positioned with the orbitomeatal line parallel to the transaxial plane of the tomograph scanner. Head position was marked, monitored throughout the scan and movement minimized using a head strap. After acquiring a CT scan for attenuation correction, approximately 150 MBq of [^18^F]-FDOPA was administered by bolus intravenous injection. PET data were acquired in 32 frames of increasing duration over the 95 min scan (frame intervals: 8 × 15 s, 3 × 60 s, 5 × 120 s, 16 × 300 s).

### Image analysis

A region-of-interest (ROI) analysis was conducted to measure Ki^cer^ (Ki in previous publications; Howes et al., 2013), using MATLAB R2018b (MATLAB, 2018), SPM12 and Piwave (Turkheimer et al., 1999). To correct for motion in the scanner, frames were realigned using the non-attenuation corrected images in combination with a level 2 order 64 Battle Lemarie wavelet filter. The transformation parameters were then applied to the corresponding attenuation-corrected images. For each subject, the movement-corrected dynamic frames were summed over time to define an individual brain PET space to which the Martinez striatal atlas (Martinez, 2003) was nonlinearly coregistered. Our primary region-of-interest was the associative striatum as this region is associated with the largest dopaminergic changes in schizophrenia, and Ki^cer^ in this region predicts improvement following antipsychotic treatment (McCutcheon et al., 2018). Data for the whole striatum and other subdivisions are reported for completeness. We used the striatal functional subdivisions (i.e. bilateral whole striatum, limbic, associative and sensorimotor subregions) defined as described by Martinez and colleagues (Martinez, 2003) together with a tracer specific template (Egerton et al., 2010; Howes et al., 2009) to create an ROI map, with the cerebellum as the reference region as defined by Egerton and colleagues (Egerton et al., 2010). Ki^cer^ was calculated for the whole striatal ROI and the bilateral functional subdivisions using the Patlak-Gjedde graphical approach adapted for a reference tissue input function (Egerton et al., 2010).

### Statistical analysis

Data were analysed using SPSS version 22 (IBM Corp, Chicago, Illinois, USA). Data normality and sphericity were assessed using the Shapiro–Wilk test and the Mauchly’s test of sphericity, respectively. Categorical clinical, demographic and experimental variables were assessed using *χ*^2^ tests, while continuous variables were assessed using *t*-tests. Effect sizes were reported using Hedge’s *g*, as it is more robust for small sample sizes.

Our first aim was to determine whether AUT00206 reduced DA synthesis capacity in the associative striatum, and whether changes in DA synthesis capacity were correlated with symptom changes based on PANSS scores. To address this, we used a paired samples *t*-test to investigate the differences in Ki^cer^ between baseline and on-treatment in the AUT00206 group in the associative striatal region. Pearson’s correlation coefficient was used to test the relationship between change in Ki^cer^ and change in symptom severity. Change was calculated by subtracting day 28 scores from baseline scores. Although the study was not powered to compare effects between placebo and AUT00206 groups on PANSS or [^18^F]-FDOPA measures, we conducted an exploratory analysis to determine whether there were group effects. This used a mixed repeated measures analysis of variance (ANOVA) with group (AUT00206 vs placebo) and time (pre- and on-treatment) to determine whether there was a main effect of group and a Group × Time interaction on striatal Ki^cer^. We used the same approach to investigate group effects on Ki^cer^ in the whole striatum and other striatal subdivisions (limbic and sensorimotor striatum). Significant interactions were explored with independent samples *t*-tests. To assess if there were significant differences in sample characteristics and scan parameters, independent samples *t*-tests were used to compare variables between patients in the AUT00206 and placebo groups. Analysis results were deemed significant if *p* < 0.05. Corrections for multiple comparisons were applied using Bonferroni corrections, where necessary.

Our second aim was to determine the test–retest reliability of [^18^F]-FDOPA imaging in patients with schizophrenia. To address this, we determined the ICCs for average Ki^cer^ values sampled from the striatum and its functional subdivisions in the placebo-treated group. We calculated the ICC using the formula defined by Shrout and collegues (Shrout and Fleiss, 1979) for case 3, which considers ‘raters’ fixed (in our case because the same PET scanner was used for all participants at all timepoints)

$$\mathrm{ICC}\;\left(3,1\right)=\frac{\left(\mathrm{BMS}\;-\;\mathrm{EMS}\right)}{\left(\mathrm{BMS}\;+\;\left(k\;-\;1\right)\;\times \;\mathrm{EMS}\right)}$$

where BMS is the subject sum of squares, EMS is the residual error sum of squares and *k* is the number of repeated sessions. This model determines the correlation between subject [^18^F]-FDOPA Ki^cer^ values between scan sessions using a two-way ANOVA with random subject effects and fixed session effects. We chose a two-way random model because the observations (Ki^cer^ values) were acquired over two sessions (test or retest scan), and this model accounts for systematic sources of variance associated with session effects (McGraw and Wong, 1996). ICC values can range from −1 to +1. A positive ICC indicates that the measures are correlated within an individual, while a negative ICC value indicates they are anti-correlated. The closer the ICC is to +1, the greater the variance is due to between-subject than within-subject variation, indicating higher reliability. Test–retest variability (VAR) was calculated as the absolute value percentage test–retest difference:

$$\mathrm{VAR}\;=\;\frac{\left|\;\left(\mathrm{retest}\;\mathrm{value}\;-\;\mathrm{test}\;\mathrm{value}\right)\;\right|}{0\mathrm{.5}\left(\mathrm{test}\;\mathrm{value}\;+\;\mathrm{retest}\;\mathrm{value}\right)}\;\times \;100$$

Variation between subjects was expressed as percentage coefficient of variation (CV):

$$\mathrm{CV}\;=\;\frac{\mathrm{SD}}{\mathrm{mean}}\;\times \;\mathrm{100}$$

## Results

### Demographics and experimental variables

A total of 24 volunteers took part in the study, of whom 20 completed both baseline and on-treatment scans (13 in the AUT00206 group and 7 in the placebo group). Four participants withdrew from the on-treatment scans due to claustrophobia. Demographic information and scan parameters are summarised in Table 1.

**Table 1.** Demographic and clinical sample characteristics.

	AUT00206		Placebo	
	Baseline, *N* = 13	On-treatment, *N* = 13	Baseline, *N* = 7	On-treatment, *N* = 7
Days between scans, Mean (SD)		19.92 (4.5) Range: 20–44 days		25.33 (10.48)Range: 16–45 days
Age (years), Mean (SD)	29.13 (6.51)	29.34 (6.62)	29.12 (10.75)	29.35 (11.72)
Weight (kg), Mean (SD)	84.43 (25.20)	85.62 (26.76)	83.02 (34.97)	88.84 (33.47)
Dose of radioactivity (Mbq), Mean (SD)	146.85 (29.58)	143.38 (11.4)	130.47 (48.95)	146.01 (39.46)
Injected mass of radiotracer (μg), Mean (SD)	1.25 (0.45)	1.26 (0.41)	1.06 (0.62)	1.22 (0.41)
SA (GBq/μmol), Mean (SD)	0.03 (0.01)	0.03 (0.01)	0.03 (0.01)	0.03 (0.01)
PANSS positive, Mean (SD)	19.86 (4.57)	16.00 (4.22)	19.14 (1.46)	17.00 (3.41)
PANSS negative, Mean (SD)	20.36 (4.20)	18.93 (5.34)	20.00 (4.16)	17.29 (2.14)
PANSS general, Mean (SD)	39.36 (4.85)	33.86 (4.63)	37.29 (4.19)	34.43 (1.71)
PANSS total, Mean (SD)	79.57 (11.71)	68.79 (10.98)	76.43 (8.10)	68.71 (6.07)
Medication				
CPZ equivalent dose/mg per day, Mean (SD)	252.6 (148.6)		181.6 (40.0)	
First-generation antipsychotic N	2		0	
Second-generation antipsychotic	6		5	
Third-generation antipsychotic	4		2	
Combination antipsychotics	1		0	

CPZ: chlorpromazine; GBq: gigabecquerel; kg: kilograms; MBq: megabecquerel; μg: microgram; mg: miligram; mm: millimetres; *N*: number; PANSS: Positive and Negative Syndromes Scale; SA: specific activity.

## The effect of AUT00206 on striatal dopaminergic function

**Figure 1.**
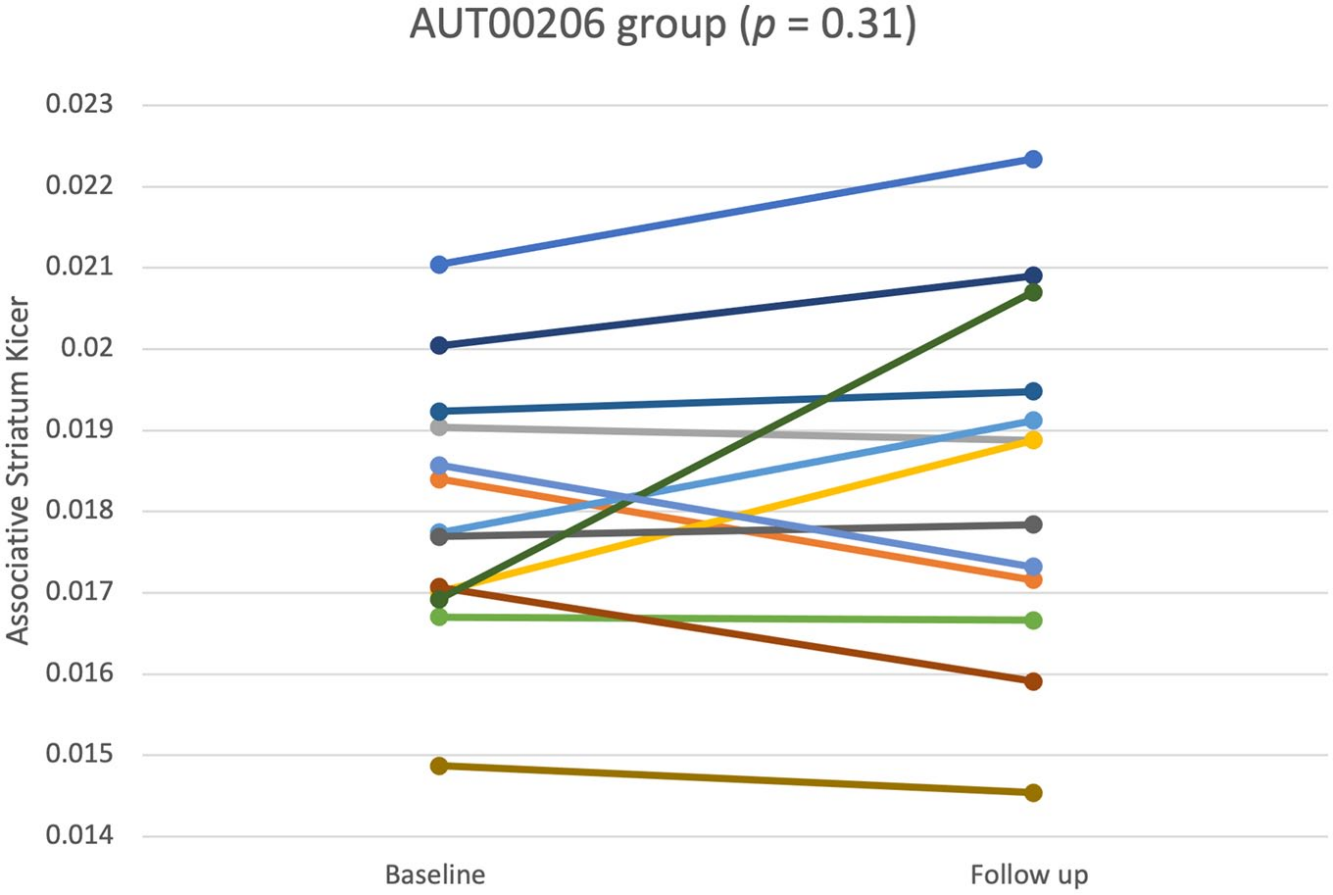
Associative striatal Ki^cer^ values at baseline and on-treatment (*M* = 19.92 days on-treatment, SD = 4.5), shown separately for each subject in the AUT00206 group. There was no significant change over time (*p* = 0.3).

Figure 1 shows the Ki^cer^ values for the associative striatum at baseline and on-treatment, in the AUT00206 group. Data were normally distributed and sphericity assumptions were met for associative striatal Ki^cer^ values. There was no significant difference between baseline and on-treatment Ki^cer^ values in the associative striatum in the AUT00206 group (*M*_baseline_ = 0.0180, SD = 0.001; *M*_on-treatment_ = 0.0184, SD = 0.002; *t*_12_ = −1.05; *p* = 0.32, 95% CI = [−0.0012, 0.0004], *g* = 0.214) or other striatal regions (see supplementary material). Similarly, there was no significant difference between baseline and on-treatment Ki^cer^ values in the associative striatum in the placebo group (*M*_baseline_ = 0.0196, SD = 0.002; *M*_on-treatment_ = 0.0198, SD = 0.002 *t*_6_ = −0.28; *p* = 0.79, 95% CI = [−0.0016, 0.0013], *g* = 0.11).

We conducted an exploratory comparison of the placebo and AUT00206 groups. This showed there was no significant effect of treatment group (*F*_1,18_ = 3.32, *p* = 0.08), time (*F*_1,18_ = 0.7, *p* = 0.41) and no significant treatment group × time interaction (*F*_1,18_ = 0.12, *p* = 0.72) on Ki^cer^ values in the associative striatum or in the other striatal regions (see supplementary material).

### Change in symptom severity and relationship to dopaminergic function

**Figure 2.**
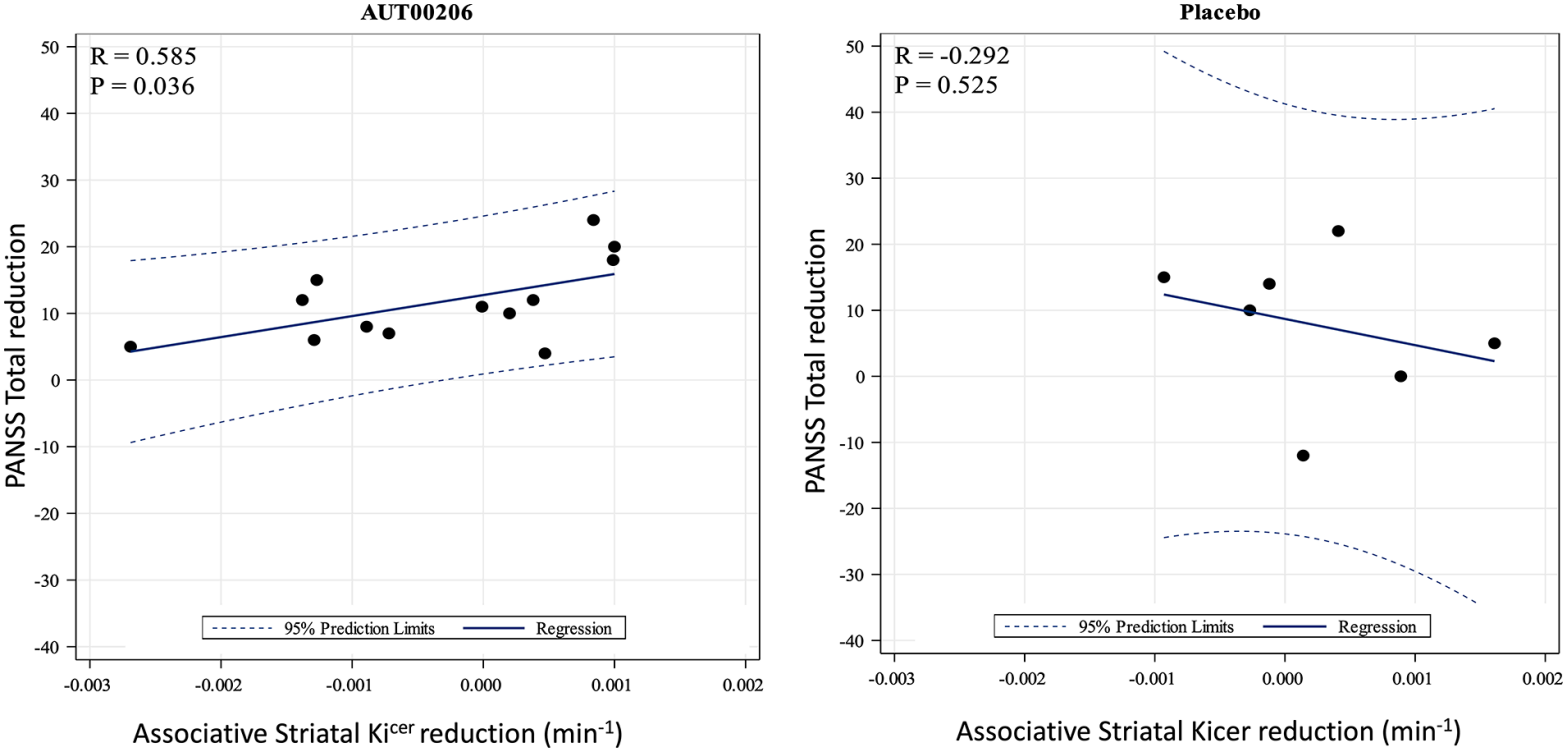
Scatter plot showing the correlation between change in associative striatal Ki^cer^ and change in PANSS total in the AUT00206 and placebo group, respectively. In the AUT00206, group greater reduction in striatal Ki^cer^ was associated with greater improvement in symptoms (*r* = 0.59, *p* = 0.04). In the placebo group, this relationship was not significant (*r* = −0.29, *p* = 0.53).

Table 1 shows the symptom ratings at baseline and on-treatment for the two groups. In the AUT00206 group, there was a significant reduction from baseline to on-treatment assessments of PANSS positive score (*t*_13_ = 4.75, *p* = 0.001, 95% CI = [2.11, 5.61], *g* = 0.782), general psychopathy score (*t*_13_ = 6.37, *p* < 0.001, 95% CI = [3.64, 7.36], *g* = 1.089) and total scores (*t*_13_ = 5.59, *p* < 0.001, 95% CI = [6.88, 14.70], *g* = 0.89), but no significant change in PANSS negative scores (*t*_13_ = 1.64, *p* = 0.48, 95% CI = [−0.45, 3.31], *g* = 0.27), although it should be noted that, in absolute terms, there were similar reductions in the placebo group as well (Table 1). There was a significant positive correlation between reduction in the associative striatal Ki^cer^ and reduction in PANSS total ratings (*r* = 0.59, *p* = 0.03, Figure 2). In exploratory analyses, we found a significant positive correlation between associative striatal Ki^cer^ change and reduction in PANSS positive ratings (*r* = 0.56, *p* = 0.04), but not for the general or negative ratings, in the AUT00206 group. For completeness, we explored the relationship between change in the other striatal regions and PANSS change, finding no significant associations (See supplementary material).

No significant changes in symptom scores were observed in the placebo group for PANSS positive (*t*_6_ = 1.54, *p* = 0.46, 95% CI = [−1.25, 5.54], *g* = 0.70), negative (*t*_6_ = 2.00, *p* = 0.36, 95% CI = [−0.61, 6.04], *g* = 0.65), general (*t*_6_ = 1.54, *p* = 0.68, 95% CI = [−1.69, 7.40], *g* = 0.80) or total (*t*_6_ = 1.81, *p* = 0.44, 95% CI = [−2.68, 18.11], *g* = 0.94) scores, and there was no significant relationship between change in symptom ratings and Ki^cer^ values in the associative or other striatal regions. In an exploratory group comparison, there was a significant effect of time (*F*_1,18_ = 36.84, *p* < 0.0001), but no significant time × group interaction for any PANSS scale.

### Test–retest reliability of [^18^F]-FDOPA in the placebo group

**Figure 3.**
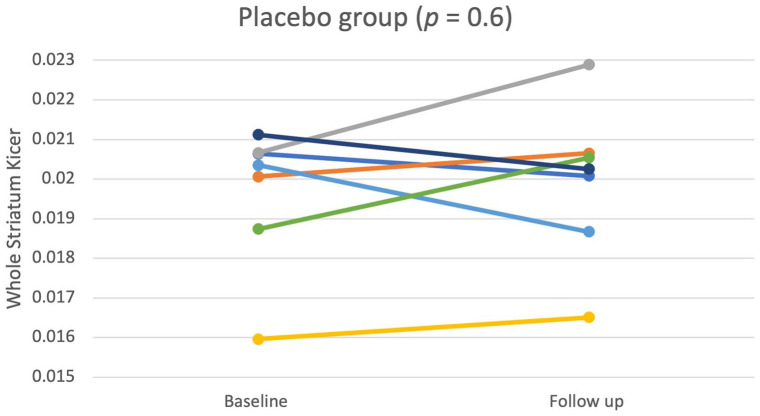
Whole striatal Ki^cer^ (min^−1^) estimates at baseline and on-treatment (placebo) timepoints (*M* = 25.33 days later, SD = 10.48) in the placebo group. There was no significant change over time (*p* = 0.6).

The mean time between baseline and on-treatment scans was 25.33 days (SD = 10.48). Figure 3 shows whole striatal Ki^cer^ values at baseline and on-treatment for each participant. There was no significant difference between the two timepoints in the striatal Ki^cer^ values (*t*_6_ = −0.55, *p* = 0.60, 95% CI = [−0.0017, 0.0010], *g* = −0.14). The Ki^cer^ values, interclass correlations and variance data are shown in Table 2.

**Table 2.** ICC, CV and VAR for the whole striatum and its functional subdivisions before and after treatment with placebo.

Region	Test Ki^cer^ (min^−1^) (*M* ± SD)	CV (%)	Retest Ki^cer^ (min^−1^) (*M* ± SD)	CV (%)	ICC	%VAR (*M* ± SD)
Whole striatum	0.0196 ± 0.0017	9.12	0.0199 ± 0.0019	9.82	0.84	1.44 ± 6.99
Sensorimotor striatum	0.0198 ± 0.0017	9.05	0.0205 ± 0.0024	12.06	0.86	3.2 ± 6.87
Limbic striatum	0.0189 ± 0.0016	8.71	0.0192 ± 0.0011	6.17	0.93	1.38 ± 4.01
Associative striatum	0.0196 ± 0.0019	9.94	0.0198 ± 0.0020	10.22	0.83	0.85 ± 7.85

CV: coefficient of variation; ICC: intraclass correlation coefficient; VAR: test–retest variability.

## Discussion

Our first main finding is that reduction in associative striatal Ki^cer^ was associated with improvement in positive and total symptoms in the AUT00206 treated group, although there was no overall group effect of AUT00206 on Ki^cer^. Our second main finding is that striatal Ki^cer^ can be measured with very good test–retest reliability (ICC > 0.83) in patients with schizophrenia over a ~1-month interval using [^18^F]-FDOPA PET imaging.

This extends the work by Egerton and colleagues (Egerton et al., 2010) in healthy volunteers to show good-to-very good test–retest reliability of [^18^F]-FDOPA PET imaging for determining Ki^cer^ in the whole striatum and its functional subdivisions to patients with schizophrenia. The ICC values we report for whole striatum and associative striatum are similar to the values reported by Egerton et al. in healthy volunteers, indicating similar reliability in patients with schizophrenia. These data support the use of [^18^F]-FDOPA PET imaging as a biomarker of DA synthesis capacity in patients with schizophrenia and suggest that it could be used to explore potential treatment effects, at least over a 1-month period.

Preclinical animal model studies suggest that Kv3 channel positive modulation might influence the function of the dopamine system given the importance of these channels for regulating the activity of subsets of neurons in the basal ganglia. Positive modulation of Kv3.1 and Kv3.2 channels on GABA neurons in the VTA and substantia nigra may regulate the firing of DA neurons in these brain areas (Parekh et al., 2018). In behavioural studies, a Kv3.1/3.2-positive modulator compound, AUT1, reduced amphetamine-induced hyperactivity in mice to a similar degree to the antipsychotic drug clozapine (Hutchison et al., 2016; Parekh et al., 2018). The primary aim of the current study was to explore the potential for the Kv3.1/3.2-positive modulator, AUT00206 to influence DA synthesis capacity, as measured by [^18^F]-FDOPA PET in patients with schizophrenia. Treatment with AUT00206 did not significantly reduce Ki^cer^. A significant reduction in symptom severity was observed in the AUT00206 but not in the placebo group, albeit the placebo group may have been underpowered to detect a significant effect.

There was a significant association between a reduction in Ki^cer^ and symptom improvement in the AUT00206 group. The apparently contradictory findings that Ki^cer^ was not significantly reduced by AUT00206 at a group level but there was a relationship between reduction in Ki^cer^ and symptom improvement at an individual level could be explained by AUT00206 acting to reduce DA synthesis capacity in some patients, who showed an improvement in symptoms, but having limited effect on DA synthesis capacity in other patients, who did not show an improvement in symptoms, resulting in an overall lack of group effect on Ki^cer^. These differences could be due to heterogeneity of pathophysiology in the patients in this study such that only a subset show an effect of AUT00206 treatment. For example, a subgroup of patients with schizophrenia have been found to not have elevated striatal [^18^F]-DOPA uptake relative to controls, which was associated with non-response to antipsychotic treatment (Demjaha et al., 2012; Jauhar et al., 2019b). In the present study, all participants were within 5 years of diagnosis and not taking clozapine, but studies indicate that about 20% of patients show treatment resistance from first episode and a further 10–15% develop it during the course of illness (Demjaha, 2018; Kane et al., 2019). Thus, it is possible that we have included some treatment-resistant patients who do not show DA dysfunction.

It should also be noted that the study was powered to detect a large effect size change in Ki^cer^, and we cannot exclude smaller effects that may nevertheless be clinically meaningful. Furthermore, AUT00206 was added to up to two antipsychotic medications in this trial, so the potential for impact of the drug on clinical symptoms might have been limited. It was notable that we did not see a relationship between change in DA synthesis capacity and symptom improvement in the placebo group, which provides some support that the association observed in the AUT00206 group was a true drug effect; however, the study was not designed or powered for a comparison with placebo. A much larger study in patients with schizophrenia is needed to test whether there is a significant difference in this relationship between AUT00206 and placebo. Future studies investigating the effects of AUT00206 as a treatment for schizophrenia may benefit from an additional control group of drug-free or drug naïve patients, where the effects of AUT00206 can be measured as a stand-alone treatment, without interactions with other psychiatric medication. Furthermore, as AUT00206 does not act directly on DA receptors, it warrants further investigations using different radioligands to investigate its effects on other systems as well.

## Conclusion

In contrast to our predictions, AUT00206 had no overall effect on DA synthesis capacity, although there was a direct relationship between reduction in DA synthesis capacity and improvement in symptoms. These findings support further investigation of the potential of the Kv3.1/Kv3.2 potassium channel modulator AUT00206 to treat schizophrenia. The test–retest reliability of [^18^F]-FDOPA PET imaging in patients with schizophrenia is very good across all functional striatal subdivisions.

## Supplemental Material

sj-docx-1-jop-10.1177_02698811221122031 – Supplemental material for The effect of AUT00206, a Kv3 potassium channel modulator, on dopamine synthesis capacity and the reliability of [^18^F]-FDOPA imaging in schizophrenia Supplemental material, sj-docx-1-jop-10.1177_02698811221122031 for The effect of AUT00206, a Kv3 potassium channel modulator, on dopamine synthesis capacity and the reliability of [^18^F]-FDOPA imaging in schizophrenia by Ilinca Angelescu, Stephen J Kaar, Tiago Reis Marques, Faith Borgan, Mattia Veronesse, Alice Sharman, Anil Sajjala, Bill Deakin, John Hutchison, Charles Large and Oliver D Howes in Journal of Psychopharmacology
