# Supplementary material for: The effect of AUT00206, a Kv3 potassium channel modulator, on dopamine synthesis capacity and the reliability of [18F]-FDOPA imaging in schizophrenia
Source: J Psychopharmacol. 2022 Sep 26;36(9):1061–9. doi: 10.1177/02698811221122031 (PMC9554157; doi:10.1177/02698811221122031)
Supplement: sj-docx-1-jop-10.1177_02698811221122031 – Supplemental material for The effect of AUT00206, a Kv3 potassium channel modulator, on dopamine synthesis capacity and the reliability of [18F]-FDOPA imaging in schizophrenia [file sj-docx-1-jop-10.1177_02698811221122031.docx]

Supplementary Material

*Additional statistical analyses*

1. *The effect of AUT00206 on striatal dopaminergic function*

An exploratory analysis investigated the effects in the whole striatum, sensorimotor and limbic striatal subdivisions. Data were normally distributed (Shapiro-Wilk test), but sphericity assumptions were not met (χ2 = 16.09, p = 0.007), so the Greenhouse-Geisser correction was used throughout the analysis. There were no significant effects of group, time or region. There was also no significant interaction effect between group x time, group x region, time x region or group x region x time.

| **Region** | **AUT**  **Baseline**  **(M ± SD)** | **AUT**  **On-treatment**  **(M ± SD)** | **Placebo**  **Baseline**  **(M ± SD)** | **Placebo**  **On-treatment**  **(M ± SD)** |
| --- | --- | --- | --- | --- |
| Whole Striatum | 0.0181 ± 0.0015 | 0.0184 ± 0.0021 | 0.0196 ± 0.0017 | 0.0199 ± 0.0019 |
| Sensorimotor Striatum | 0.0182 ± 0.0015 | 0.0188 ± 0.0021 | 0.0198 ± 0.0017 | 0.0205 ± 0.0024 |
| Limbic Striatum | 0.0179 ± 0.0013 | 0.0179 ± 0.0017 | 0.0189 ± 0.00116 | 0.0192 ± 0.0011 |

Further t-tests showed that there were no differences between baseline and follow-up Ki^cer^ in the AUT00206 group in the following regions: total striatum (t_12_ = -1.12, p=0.28), sensorimotor striatum (t_12_ = -1.56, p=0.14), limbic striatum (t_12_ = -0.15, p=0.88). The same held true in the placebo group: total striatum (t_6_ = -0.55, p=0.6), sensorimotor striatum (t_6_ = -1.26, p=0.25), limbic striatum (t_6_ = -0.83, p=0.44).

1. *Change in symptom severity and relationship to dopaminergic function*

In the AUT00206 group, there were no significant correlations between change in PANSS scores and change in Ki^cer^ values in the whole striatum, sensorimotor or limbic striatum.

| **AUT00206 group** | | | | |
| --- | --- | --- | --- | --- |
|  | PANSS Positive | PANSS Negative | PANSS General Psychopathy | PANSS Total |
| Whole striatum | r = 0.25, p = 0.41 | r = 0.20, p = 0.51 | r = 0.28, p = 0.34 | r = 0.32, p = 0.28 |
| Sensorimotor striatum | r = 0.14, p = 0.63 | r = 0.03, p = 0.9 | r = 0.25, p = 0.42 | r = 0.21, p = 0.49 |
| Limbic striatum | r = 0.15, p = 0.63 | r = -0.05, p = 0.85 | r = 0.1, p = 0.74 | r = 0.09, p = 0.75 |

In the placebo group, there were also no significant correlations between change in PANSS scores and change in Ki^cer^ values in the whole striatum, sensorimotor or limbic striatum.

| **Placebo group** | | | | |
| --- | --- | --- | --- | --- |
|  | PANSS Positive | PANSS Negative | PANSS General Psychopathy | PANSS Total |
| Whole striatum | r = -0.24, p = 0.6 | r = 0.36, p = 0.93 | r = -0.28, p = 0.53 | r = -0.19, p = 0.68 |
| Sensorimotor striatum | r = -0.28, p = 0.53 | r = 0.04, p = 0.93 | r = -0.33, p = 0.47 | r = -0.23, p = 0.63 |
| Limbic striatum | r = -0.34, p = 0.45 | r = -0.21, p = 0.64 | r = 0.29, p = 0.52 | r = -0.14, p = 0.75 |


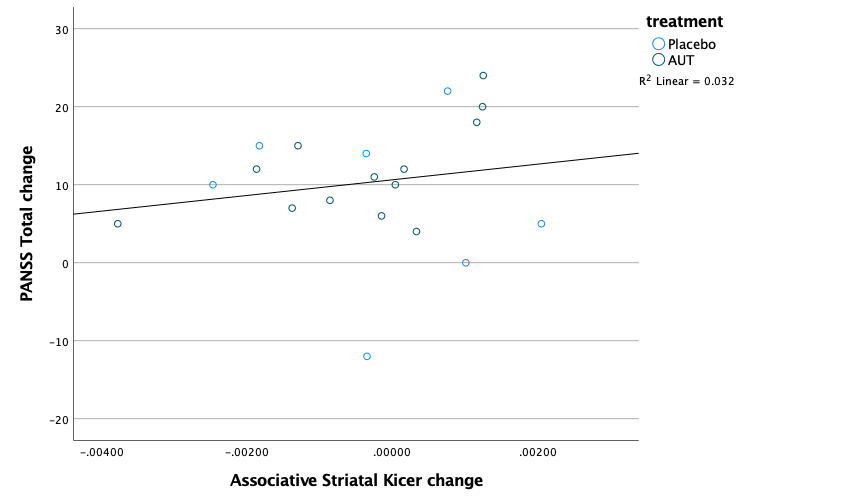


Figure 4. Relationship between Ki^cer^ changes in the Associative Striatum and PANSS Total score changes across both placebo and AUT groups (p=0.45)
